# Simulation Based Evaluation of Time Series for Syndromic Surveillance of Cattle in Switzerland

**DOI:** 10.3389/fvets.2019.00389

**Published:** 2019-11-05

**Authors:** Céline Faverjon, Sara Schärrer, Daniela C. Hadorn, John Berezowski

**Affiliations:** ^1^Vetsuisse Faculty, Veterinary Public Health Institute, University of Bern, Bern, Switzerland; ^2^Federal Food Safety and Veterinary Office, Bern, Switzerland

**Keywords:** syndromic surveillance, Holt-Winters, EWMA, syndrome selection, time series

## Abstract

Choosing the syndrome time series to monitor in a syndromic surveillance system is not a straight forward process. Defining which syndromes to monitor in order to maximize detection performance has been recently identified as one of the research priorities in Syndromic surveillance. Estimating the minimum size of an epidemic that could potentially be detected in a specific syndrome could be used as a criteria for comparing the performance of different syndrome time series, and could provide some guidance for syndrome selection. The aim of our study was to estimate the potential value of different time series for building a national syndromic surveillance system for cattle in Switzerland. Simulations were used to produce outbreaks of different size and shape and to estimate the ability of each time series and aberration detection algorithm to detect them with high sensitivity, specificity and timeliness. Two temporal aberration detection algorithms were also compared: Holt–Winters generalized exponential smoothing (HW) and Exponential Weighted Moving Average (EWMA). Our results indicated that a specific aberration detection algorithm should be used for each time series. In addition, time series with high counts per unit of time had good overall detection performance, but poor detection performance for small epidemics making them of limited use for an early detection system. Estimating the minimum size of simulated epidemics that could potentially be detected in syndrome TS-event detection pairs can help surveillance system designers choosing the most appropriate syndrome TS to include in their early epidemic surveillance system.

## Introduction

Early warning systems are critically important for controlling emerging or reemerging diseases. Dealing with a disease epidemic in its early stages is easier and more economical than dealing with an epidemic that has become large and widespread (1, 2). Traditional passive early detection systems rely on reports submitted to veterinary public health authorities by various healthcare stakeholders when they observe suspect cases in the field. This surveillance activity covers a large part of the animal population and the costs associated with data collection and analysis are relatively low (3–5). However, the performance of these passive surveillance systems suffers from frequent under-reporting due to the lack of stakeholder awareness, especially regarding emerging diseases, and fear of the consequences of reporting a disease occurrence (4, 5). To enhance traditional passive surveillance systems, real-time or near real-time surveillance systems have been developed. These systems, commonly called syndromic surveillance (SyS) systems (6), are based on pre-diagnostic often unspecific routinely collected data which is available prior to laboratory confirmation of the causative agent of an epidemic. A great variety of data can be used for syndromic surveillance (e.g., laboratory requests, milk production, Google queries, and many others). These data are converted to time series (TS) for monitoring and are referred to as syndromes (6).

Constant improvements in data science and computer technology have favored the development and implementation of SyS systems by facilitating data acquisition, and analysis. The number of operational SyS systems has constantly increased during the last decades in both human and veterinary medicine (7, 8). By simultaneously assessing information from different data sources related to different populations and/or symptoms, one can improve epidemic detection and in particular, the sensitivity and the specificity of epidemic detection (8). Choosing the syndrome TS to monitor in a SyS system is not an easy or straightforward process. Defining which syndromes to monitor in order to maximize detection performance is very challenging and has been recently identified as one of the research priorities in SyS (9). This is especially true when data can be subdivided into many syndrome classifications or definitions (9), or when the objectives of surveillance are unspecific. For these reasons selection of syndrome TS should be guided by data characteristics including representativeness and by the objectives of the surveillance system (10). However, when the objectives are broad, for example to detect not only known diseases of interest, but also new, emerging, exotic or unknown endemic diseases, they are of little help for selecting the most appropriate syndrome TS.

In operational SyS systems, syndrome TS are monitored with automated aberration detection algorithms in order to detect unexpected changes that could potentially be caused by an epidemic. A useful criterion for selecting a specific syndrome TS for a SyS system is an assessment of the nature of the change that can be detected. Any syndrome TS that is monitored with any aberration detection algorithm should be able to detect a sudden and very large variation in the number of cases reported. However, detecting a large change in a syndrome TS is of little interest for surveillance if the aim is early detection of disease epidemics. In this case detecting small changes in a time series which may represent the onset of an epidemic is of greater importance. Changes in syndrome TS should be detected with a high degree of certainty and as soon as possible after the epidemic has started. Estimating the minimum size of an epidemic that could potentially be detected in a syndrome TS may serve as a useful criteria for comparing syndrome TS performance and provide guidance for their selection.

In Switzerland, a cattle disease SyS system is currently being designed to meet the goals of the “Swiss Animal Health Strategy 2010+,”^1^ which aims to maintain and improve the high standard of animal health in the country. The purpose of the SyS system is to detect abnormal health events such as disease epidemics occurring in the Swiss cattle population by monitoring syndrome TS extracted from a central database maintained by the Federal Food Safety and Veterinary Office (FSVO). The objective of our study was to evaluate different syndrome TS as candidates for inclusion in the system. Data quality and population coverage should be always carefully assessed before including a TS into a SyS system (10). However, evaluating these criteria was not the purpose of our study and these characteristics are only briefly presented and discussed in this paper. Since the goal of SyS is early detection of epidemics, our study focused on estimating the minimum size of simulated epidemic that can be detected in syndrome TS-event detection pairs, as a criterion for inclusion in a SyS system. To standardize the comparisons between syndrome TS-event detection pairs, we created a standard set of simulated epidemics of various shapes and sizes and used this standard set to compare the performance of all syndrome TS-event detection pairs. Our study objective differs from other studies that focus on evaluating the performance of event detection algorithms only. For practical purposes, the combined performance of an event detection algorithm operating on a specific syndrome TS should be more useful to surveillance system designers.

## Materials and Methods

### Data Sources and Associated Time Series

Three databases containing data from the Swiss national cattle population were used: (1) the *Swiss Animal Movement Database* (AMD), (2) the database owned by the *Association of Swiss Cattle Breeders* (ASR), and (3) the *Swiss Laboratory Information System* (ALIS). The AMD has been studied and reported to have potential value for SyS because of its relatively high quality in terms of population representativeness and reporting timeliness (11). The other two databases contain laboratory test orders (ALIS) and clinical data collected by farmers (ASR).These two databases haven't been investigated in Switzerland, but similar data have been reported to be of value for SyS in others countries (8).

The AMD contains data on cattle mortalities, including stillbirths, reported by farmers to the Swiss national system for the identification and registration of cattle. All reported on-farm deaths and stillbirths for the period from January 1st 2009 to September 28th 2016 were extracted from the AMD. Since the reporting of on-farm deaths was mandatory, we can assume a high population coverage from this source over this period excepting for stillbirths. Stillbirths were defined as non-living fetuses expelled before the end of gestation, or calves born dead within 24 h following birth since mid-2014. Before that date, no official definition of a stillbirth existed in Switzerland. It is not mandatory to report every stillbirth to the AMD and the population coverage of this syndrome TS is unknown at the time of writing. Four syndrome TS were created from the AMD database. One was based on stillbirths (*AMD_stillbirth*) and three were based on categories of on-farm deaths defined according to the age at death: up to 6 months old (*AMD_mortality_calves*), 6 months−2 years (*AMD_mortality_young*), and more than 2 years *(AMD_mortality_adults*).

The *ASR* (http://asr-ch.ch/en/asr/) is the private umbrella organization of the Swiss cattle breeding organizations. Beginning in 2013 the ASR developed and implemented a database containing cattle illness diagnoses reported by farmers and veterinarians. All cases were reported using a coding system with four levels ranging from least specific (i.e., organ affected) to most specific (e.g., infectious agent isolated). Data were available for the most common cattle breeds in Switzerland: Braunvieh, Fleckvieh, and Holstein, which represent the majority of the Swiss dairy cattle population. No data about beef cattle were available. The timeliness of reporting to this database is unknown. Data were available from January 1st, 2014 to December 31st, 2016. Three syndrome TS were created based on the age category of diseased animal: abortions (*ASR_abortion*), diseased calves (*ASR_calves*), and diseased adults (*ASR_adults*). In the ASR database calves are defined as cattle up to 6 months of age. Abortions are defined as calves born dead, or born alive but having died within the first 24 h of life. The syndrome TS *ASR_calves* and *ASR_adults* were each split into three syndrome TS based on the most frequent diagnostic classification found in the database: gastrointestinal symptoms (i.e., *ASR_GI_calves* and *ASR_GI_adults*), respiratory symptoms (i.e., ASR_*RESPI_calves* and *ASR_RESPI_adults*), and cattle having a classification of “other” in the ASR classification schema (i.e., *ASR_OTHER_calves* and *ASR_OTHER_adults*). The category “other” encompasses various unspecific symptoms such as fever, anorexia, changing behavior or reduced production. The precise coverage of the dairy cattle population by the ASR is unknown but it is expected to be high.

The ALIS database contains data from laboratory tests performed by the 25 accredited laboratories involved in the diagnosis of epizootics in Switzerland on behalf of the FSVO. All laboratory tests performed for the 70 notifiable epizootics of interest in Switzerland are collected in ALIS. The reporting timeliness (time between the sampling date and the date when the sample was received by a laboratory) was on average of 1 day. Data were analyzed from November 1st, 2013 to July 27th, 2016. All laboratory tests performed for mandatory reasons without any clinical suspicion were excluded (e.g., mandatory surveillance programs, importation, vaccination, research activities). One syndrome TS was created containing counts of stillbirth samples sent to the accredited laboratories *(ALIS_abortion)*. Two additional syndrome TS were created from samples sent to the accredited laboratories because of clinical suspicion of two diseases of interest in Switzerland: bovine viral diarrhea (*ALIS_BVD*) and infectious bovine rhinotracheitis (*ALIS_IBR*). Suspicious cases were individual cattle and they were always confirmed (or negated) with an FSVO approved laboratory test.

**Figure 1:**
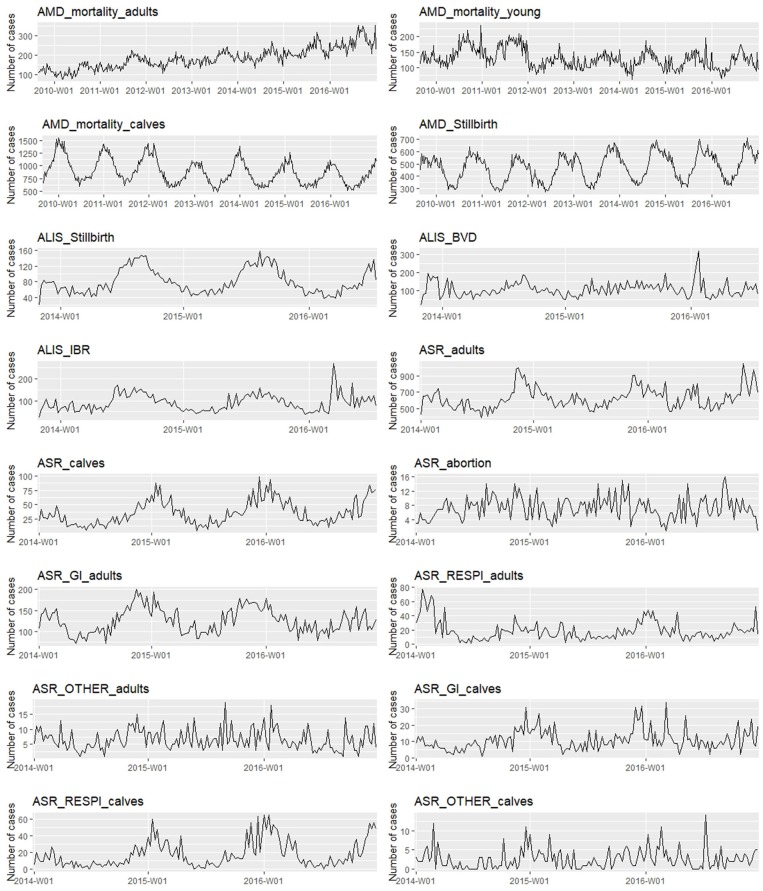
Weekly syndrome time series extracted for the study (abnormal peaks have been removed).

In total, data for 16 syndrome TS were extracted from the 3 databases and converted to weekly syndrome TS (see Figure 1).

### Time Series Analysis and Preprocessing

To the best of our knowledge there were no epidemics reported in the target cattle population during the study period. However, there was considerable variation in the data that was known to be caused by non-epidemic events. Because the historical baselines in our study were very short, extreme outliers had a substantial effect the HW models, resulting in poor TS predictions. Extreme single time point aberrations were removed in order to obtain aberration-free historical baseline data that improved the prediction performance of our models and the performance of aberration detection algorithms (12–14). We chose a manual approach for outlier removal in order to preserve as much of the natural variation as possible in the data. We examined each syndrome TS visually and manually removed only the most extreme peaks. Extreme peaks were defined as weeks where the number of reported cases equaled at least two times the number of reported cases in the neighboring weeks. Once extreme peaks were identified, they were investigated in more detail to determine if they were associated with a specific health related event or not. Peaks that were associated with health related events were considered abnormal. They were removed from baseline syndrome TS and replaced by the weekly average of the 10 previous time points. The 10 week average was used as it has been reported to provide the best prediction performance for HW models. Extreme peaks that were not associated with health related events were considered part of the normal variation and left in the baseline syndrome TS. In total there were 7 abnormal values identified, 1 week in ALIS_abortion because of suspicions of Neosporosis, and 6 weeks in ALIS_IBR likely because IBR suspect cases were identified and this may have increased veterinarian awareness of the disease, causing them to increase IBR sample submission. The best HW models were evaluated using the autocorrelation and partial autocorrelation functions of the residuals (ACF and PACF, respectively) (15) and the root-mean-squared error (RMSE) (16). RMSE is a measure of the difference between the values predicted by a model and the values actually observed from the environment that is being modeled. We calculated RMSE for the differences between the observations and the predicted values within both the training period (RMSE_t_) and the validation period (RMSEv). In both cases, the predictive performance of the HW model are better when the criterion is lower.

Regression models were fitted to the syndrome TS to estimate the linear trend and annual seasonality. Poisson and negative-binomial regression models were fit to the syndrome TS for the full time period available for each syndrome TS. Likelihood ratio tests were used to test for the significance of each predictor at a statistical significance level of 5%. Syndrome TS were then characterized using 4 parameters adapted from Choi (17):

1. Length of the historical baseline: long when >3 years, short when ≤ 3 years;
2. Linear trend: positive, negative, or none;
3. Annual seasonality: none when no monthly effect identified. When there was a monthly effect, the strength of the seasonality was assessed based on the value of F_s_ (18) calculated as equal to 1–Var (R)/Var(S + R). Var (R) is the variance of the remainder component of the syndrome TS and Var (S + R) is the variance of the detrended syndrome TS. Seasonality was considered to be weak when F_s_ was below 0.5, and strong when it was ≥0.5;
4. Mean and standard deviation (SD) of the weekly counts, and corresponding coefficient of variation (CV).

### Aberration Detection Algorithms

Two different aberration detection algorithms were compared: Holt–Winters generalized exponential smoothing (HW) (19, 20) and Exponential Weighted Moving Average (EWMA) (21, 22). Ten different detection limits, or alarm thresholds, were tested for both algorithms. To avoid contamination of the baseline with cases from gradually increasing epidemics, a guard-band of 2 weeks was used between the baseline and the current value being evaluated.

#### Holt-Winters

HW is a triple exponential smoothing method which involves exponentially decreasing the weights of observations over time, such that oldest observations have the smallest weight. The forecast is continuously revised according to the most recent observations. HW incorporates three components: a level term, a trend term, and a seasonality term, respectively, defined by the smoothing constants α, β, and γ. HW can be applied to raw time series containing trend and seasonality. All the data available before 31-12-2015 were used for model training. The data available after December 31st, 2015 were used for model validation and for the estimation of model prediction performance. The training data contained data for periods from 2 to 7 years and the validation data contained data for periods from 7 to 12 months, depending on the length of syndrome TS. Optimal HW parameters were determined through minimization of the squared prediction error (23). Model fit was evaluated using the autocorrelation and partial autocorrelation functions of the residuals (ACF and PACF, respectively), normality Q-Q plot, and the root-mean-squared error (RMSE). ACF is the linear dependence of a variable on itself at two points in time and PACF is the autocorrelation between two points in time after removing any linear dependence between them (15). ACF and PACF were used to find any remaining repeated patterns in the model residuals. RMSE is a measure of the difference between the values predicted by a model and the values actually observed in the real data (16). This criterion was calculated for the differences between the observed and the predicted values within both the training period (RMSE_t_) and the validation period (RMSE_v_). In both cases, the predictive performance of the model were better when the RMSE was lower. The alarm thresholds tested for evaluating event detection performance were based on constant values multiplied with the standard error of the predicted value for each week (21, 22). The following constant values were used: 0.05, 0.1, 0.25, 0.5, 1, 1.5, 2, 2.5, 3, 3.5, 4, 4.5.

#### EWMA

EWMA is the simplest form of exponential smoothing and it relies on cumulative differences between observed data in a time window and a threshold. It is based on the equation: E_t_ = (1 – λ)^t^ E_0_ + Σ_ti = 1_ (1 – λ)^t^ λI_t_ where λ is the smoothing parameter (>0) that determines the relative weight of current data in relation to past data, I_t_ is the observed value at time t, and E_0_ is the starting value. EWMA is recommend only for stationary and normally distributed TS (21, 22). A 1 week differencing (i.e., computation of the difference between consecutive weekly time points) was used to remove the largest temporal effects present in the raw data. The differenced residuals were saved as a new TS. Autocorrelation and normality in the TS of residuals were assessed using ACF, PACF and normality Q-Q plot in order to evaluate whether pre-processing enabled transformation of the weekly auto-correlated TS into stationary and normally distributed TS. EWMA was then applied to the residual TS using a smoothing parameter λ of 0.2. The same constant values that were used for HW were also used for calculating the alarm thresholds for the EWMA algorithm.

### Data Simulation

We simulated epidemic-free baseline TS for each syndrome using the model predictions obtained from the best fitting HW model that was developed using the data available before 2016. The mean fitted value for each week of the year was used as the mean of weekly Poisson distributions (one for each week of the year). We then randomly sampled from each weekly Poisson distribution to simulate 300 epidemic-free baseline TS for each syndrome.

Twenty five different epidemics types were simulated based on five different epidemic shapes and five epidemic magnitudes (see Table 1 and Appendix 1). Five epidemic shapes representing different temporal progressions of an epidemic within a population were created, based on (24, 25): single spike, flat, linear, exponential, and log normal. The length of all simulated epidemics was fixed at 12 weeks except for the epidemic shape “single spike” which lasted only 1 week. We choose an epidemic length of 12 weeks (3 months) because we were interested in evaluating the syndrome time series for early epidemic detection. We were not interested in alarms after 12 weeks as in our opinion these would not qualify as early detection. Epidemic magnitude represents the severity of the epidemic and was defined as the maximum number of additional cases added to the weekly epidemic-free baseline during the epidemic time period. In this context a case equals a diseased animal reported in the data. Five different epidemic magnitudes were tested: 25, 50, 150, 300, and 500 corresponding, respectively, to very small, small, medium, large, and very large epidemics. The magnitudes represented the maximum number of extra cases added per week to the epidemic-free baseline during the epidemic time period. As an example, for an epidemic with a magnitude of “150”; 150 cases were added to the epidemic-free baseline at the peak of the epidemic which was on week number 12 of the epidemic time period. Smaller numbers of epidemic cases were also inserted for each of the 11 weeks prior to the epidemic peak. The exact number of extra cases added to the epidemic-free baseline for the pre-peak weeks was calculated according to the different epidemic shapes (see Table 1 for details). For the “single spike” epidemic, which lasted 1 week, the epidemic magnitude always represented the total number of cases in the epidemic.

**Table 1:** Methods for epidemic simulation adapted from Dórea et al. (24) and Lotze et al. (25).

**Epidemic shape**	**Method used for epidemic simulation**
Single spike	Epidemic length was always 1 week and on that week, extra_k_ equals the epidemic magnitude (25, 50, 150, 300, or 500).
Flat	Extra_k_ always equals to the epidemic magnitude (25, 50, 150, 300, or 500) for the 12 weeks of the epidemic time period.
Linear	Extra_k_ increases linearly until it reaches a maximum value equal to the epidemic magnitude on week 12 of the epidemic time period. Example for an epidemic magnitude of 25, starting on week *i*, extra_i+k_ equals successively 2, 4, 6, 8, 10, 12, 15, 17, 19, 21, 23, 25 for *k* varying from 1 to 12.
Exponential	Extra_k_ increases exponentially until it reaches a maximum value equal to the epidemic magnitude on week 12 of the epidemic time period. For the duration of 12 weeks, this was achieved by assigning the maximum number of extra cases (i.e., the epidemic magnitude) to the last week of the epidemic time period, and dividing each week by 1.3 to obtain the value for the preceding week. Example for an epidemic magnitude of 25, starting on week *i*: extra_i+k_ is calculated as follow: extra_i+12_ = 25, extra_i+11_ = extra_i+12_/1.3, extra_i+10_ = extra_i+11_/1.3, etc. extra_i+k_ equals thus successively 1, 2, 2, 3, 4, 5, 7, 9, 11, 15, 19, 25 for *k* varying from 1 to 12.
Lognormal	Extra_k_ increases following a log normal curve until it reaches a maximum value equal to the epidemic magnitude on week 12 of the epidemic time period. The percentage of increase in week *k* of the epidemic time period equals the x^ieme^ percentile of a log normal distribution [lognormal (4, 0.3)]. Example for an epidemic magnitude of 25, starting on week *i*, extra_i+k_ equals successively: 6, 10, 12, 14, 16, 17, 19, 20, 21, 22, 23, 25 for *k* varying from 1 to 12.

*count_i_, The number of cases in week i which equals fit_i_ + extra_i_; fit_i_, the mean fitted value of the Poisson distributions that characterized this week i of the baseline; extra_k_, number of cases added due to the epidemic on week k*.

**Figure 2:**
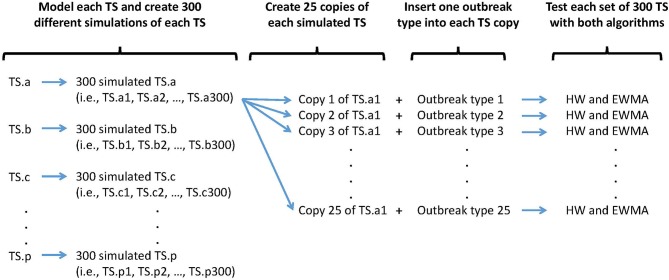
Synthetic outbreak and baseline simulation process. An outbreak type is defined by a specific shape (i.e., single spike, flat, linear, exponential, or lognormal) and a specific size (i.e., very small, small, medium, large, very large).

Three hundred epidemics of each type were simulated and randomly inserted within the 300 simulated baselines. Only one epidemic was inserted per simulated baseline to avoid epidemic overlap. Each simulated baseline was used 25 times to detect 25 different epidemics types characterized by different epidemic shapes and magnitudes. In other words, to assess the algorithms and time series capacities to detect a certain epidemic shape of a certain magnitude, 300 different simulated baselines were used. The process and resulting syndrome TS are presented in Figure 2.

### Detection Performance Estimation

We calculated sensitivity (*Se*) based on the number of epidemics detected out of all inserted epidemics. An epidemic was detected when it triggered at least one true alarm, defined as a week that produced an alarm within an epidemic period. *Se* was calculated as:

(1) $$\begin{array}{r} \text{Se}=\text{Epidemics detected}/\text{Total number of epidemics inserted} \end{array}$$

We also calculated the specificity (*Sp*), the positive predictive value (*PPV*) and the negative predictive value (*NPV*) as:

(2) $$\begin{array}{r} \text{Sp}=\text{TN}/\left(\text{TN}+\text{FP}\right) \end{array}$$

(3) $$\begin{array}{r} \text{PPV}=\text{TP}/\left(\text{TP}+\text{FP}\right) \end{array}$$

(4) $$\begin{array}{r} \text{NPV}=\text{TN}/\left(\text{TN}+\text{FN}\right) \end{array}$$

where TP is the number of true positive alarms (i.e., alarms raised on a week which is part of an epidemic period), TN the number of true negative alarms, FP the number of false-positive alarms (i.e., alarms raised on a week which is not part of an epidemic period), and FN the number of false negative alarms.

A receiver-operating characteristic (ROC) curve was generated and, assuming equal costs for false negative and false positive alarms, we graphically defined the optimal alarm threshold where *Se* and *Sp* were at a maximum. The timeliness of the first alarm raised during an epidemic time period was computed. Detection timeliness was the time lag (in weeks) between the start of the epidemic and the first alarm. A value of 1 meant that the first alarm was raised during the second week of the epidemic. Single spikes were excluded from the computation of the detection timeliness as they always lead to detection on the first, and only week of the epidemic. The cumulative number of cases occurring because of the epidemic when the first alarm was raised (*cum_cases*) was also calculated. We calculated Spearman's nonparametric correlation coefficients (ρ) to test the association between the size of the syndrome TS (in terms of counts per week) and the detection performance of the syndrome TS.

### Software Implementation

All statistical analyses were implemented in R x64 version 3.4.1 (26). Dynamic regression was performed with the functions glm (package “stats”), glm.nb [package “MASS” (27)], and stl [package “forecast” (28)]. The stl function was also used to estimate the detrended and remainder component of each syndrome TS and calculate the strength of the seasonality. The expected numbers of counts at time t for HW were estimated with the predict functions of the “forecast” packages. EWMA and HW aberration detection algorithms were executed using the package “Vetsyn” package (29).

## Results

### Time Series Description

Seven of the syndrome TS in this study had a linear trend and all 16 syndrome TS had seasonality, however, the syndrome TS peaked in different seasons (see Table 2 and Figure 1). The main differences between these syndrome TS were the length of historical data available, which ranged from slightly less than 3 years to more than 7 years; and the average number of reports per week that varied from a low of 2.6 for *ASR_OTHER_calves* to a high of 891 for *AMD_mortality_calves*. An interesting observation was that in general the coefficient of variation (CV) was greater for syndrome TS with smaller average weekly counts. All syndrome TS with counts >100 counts/week had on average a CV of <0.30. All syndrome TS with average weekly counts around or <100 had CV values of >0.39.

**Table 2:** Time series characteristics.

**Time series**	**Length of the historical data**	**Trend**	**Seasonality (F_**s**_)**	**Mean weekly count (SD)**	**Coefficient of variation (CV)**
AMD_mortality_calves	2009–2016	–	0.91–strong	891.8 (241)	0.27
ASR_adults	2014–2016	0	0.74–strong	629 (129.2)	0.20
AMD_Stillbirth	2009–2016	+	0.92–strong	466 (108)	0.23
AMD_mortality_adults	2009–2016	+	0.54–strong	170 (42)	0.24
AMD_mortality_young	2009–2016	–	0.58–strong	130.4 (31)	0.23
ASR_GI_adults	2014–2016	0	0.74–strong	125 (29.3)	0.23
ALIS_BVD	2013–2016	0	0.38–weak	105.4 (41.9)	0.39
ALIS_IBR	2013–2016	0	0.54–strong	94.8 (39.5)	0.41
ALIS_abortion	2013–2016	0	0.89–strong	79.6 (31.7)	0.40
ASR_calves	2014–2016	+	0.75–strong	36 (20.9)	0.58
ASR_RESPI_adults	2014–2016	0	0.46–weak	19 (14)	0.73
ASR_RESPI_calves	2014–2016	+	0.69–strong	17.7 (15.8)	0.89
ASR_GI_calves	2014–2016	0	0.50–strong	11 (6.3)	0.57
ASR_Abortion	2014–2016	0	0.36–weak	7 (3.1)	0.44
ASR_OTHER_adults	2014–2016	0	0.36–weak	6 (3.5)	0.58
ASR_OTHER_calves	2014–2016	0	0.36–weak	2.6 (2.6)	1

*Length of the historical baseline, trend (positive, negative, none), seasonality [none, weak (F_s_ < 0.5), strong (F_s_ ≥ 0.5)]. TS were ordered according to the weekly average number of notifications from the largest (top row) to the smallest (bottom row)*.

### Time Series Modeling and Preprocessing

The fitting and prediction performance of HW for each syndrome TS is shown in Appendix 2.A. The HW method removed most of the autocorrelations present in the raw data but sporadic autocorrelations remained. The HW method correctly predicted the values of the validation dataset. However, the accuracy of the predictions varied a lot depending on the syndrome TS evaluated (see Appendix 2.A).

Autocorrelation function plots of the 1 week differencing for the 16 syndrome TS are shown in Appendix 2.B. All the syndrome TS had similar results and 1 week differencing removed most of the autocorrelations present in the raw syndrome TS (Appendix 2.C). Some autocorrelations remained, especially at lag 1. One-week differencing did not remove this residual autocorrelation and even produced some residual TS with a higher number of significant autocorrelations. The syndrome TS created with 1 week differencing were used to implement the aberration detection algorithm EWMA.

### Detection Performance

#### Comparing Algorithms

As expected, both aberration detection algorithms performed better with large epidemics as compared to small epidemics (i.e., higher sensitivity, specificity, and detection timeliness). Flat epidemics were always detected with higher sensitivity, specificity, and timeliness than log normal and linear increases. Single spikes and exponential increases had the worst performance and were the epidemic shapes most difficult to detect for both algorithms. There was no difference in the performance of the two algorithms for different epidemic shapes (see Appendix 3, Figures 1, 2).

**Figure 3:**
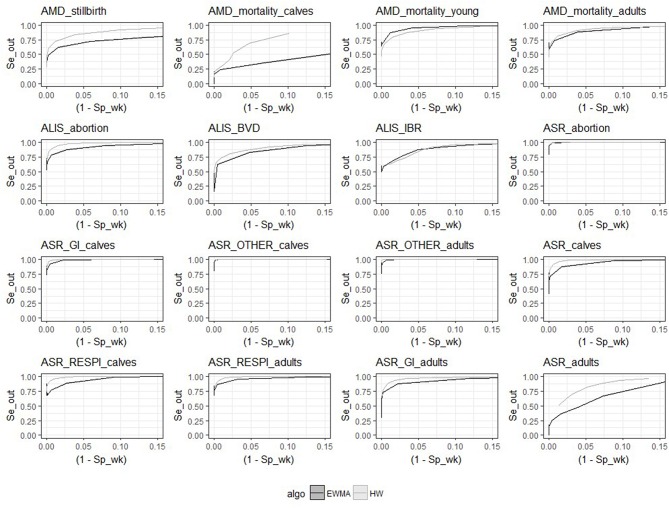
Receiver-operating characteristic (ROC) curves for the 16 syndrome TS and the 2 aberration detection algorithms (all epidemic sizes and shapes are collated).

**Figure 4:**
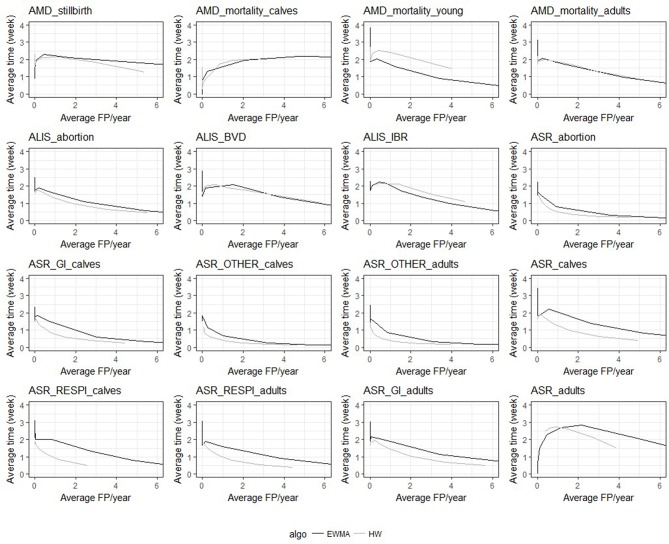
Overall average detection timeliness (week) and corresponding average number of expected false positive alarms (FP) per year for the two algorithms and the 16 TS (all epidemic sizes and shapes are collated).

Despite the similarities mentioned above, the two algorithms had different relative performance depending on the syndrome TS. The Holt-Winters algorithm outperformed EWMA for 12 syndrome TS: *AMD_stillbirth, AMD_mortality_calves, AMD_mortality_adults, ASR_OTHER_adults, ALIS_abortion, ALIS_BVD, ASR_GI_calves, ASR_calves, ASR_RESPI_calves, ASR_RESPI_adults, ASR_GI_adults*, and *ASR_adults*. The EWMA algorithm outperformed HW for only 2 syndrome TS: *AMD_mortality_young*, and *ALIS_IBR*. Both algorithms had equivalent sensitivity and specificity for 2 syndrome TS: *ASR_abortion*, and *ASR_OTHER_calves* (see Figure 3). The HW algorithm had equivalent or a better balance between detection timeliness and the average number of false positive alarms than EWMA in most syndrome TS (see Figure 4). However, EWMA had better timeliness for *ALIS_IBR* and *AMD_mortality_young*.

The HW algorithm performed better detection for the following time series: *AMD_stillbirth, AMD_mortality_calves, AMD_mortality_adults, ASR_OTHER_adults, ALIS_abortion, ASR_GI_calves, ASR_calves, ASR_RESPI_calves, ASR_RESPI_adults, ASR_GI_adults*, and *ASR_adults*. This algorithm was thus considered to be the optimal algorithm for these syndrome TS, and was used for all further analyses of these syndrome TS. Whereas, the EWMA algorithm demonstrated better detection performance with the syndrome TS: *AMD_mortality_young* and *ALIS_IBR*. The detection timeliness of *ALIS_BVD* were equivalent for both HW and EWMA, but the overall sensitivity and specificity was slightly better with HW. HW was chosen as the most appropriate aberration detection algorithm for *ALIS_BVD*.

#### Comparing Syndrome Time Series

The optimal alarm threshold for the optimal algorithm previously selected for each syndrome TS was estimated as the alarm threshold where *Se* and *Sp* were at a maximum. This assumes equal costs for false negative and false positive alarms. The detection performances obtained at the optimal alarm thresholds are summarized in Tables 3, 4, and in Appendix 4.

**Table 3:** Global Detection performance obtained with the optimal algorithm at the optimal alarm threshold.

**Time series**	**Mean weekly count**	**Optimal algorithm**	**Optimal alarm threshold**	**Se**	**Sp**	**PPV**	**NPV**	**FP per year**	**T**
AMD_mortality_calves	891.8	HW	0.05	85.9 (85.1–86.7)	88.8 (89.2–90.5)	44.9	84.8	4.0	2.0
ASR_adults	629	HW	0.5	93.4 (92.8–94)	90.4 (89.8–91.1)	56.5	88.3	3.8	2.1
AMD_Stillbirth	466	HW	0.5	92.4 (91.8–93.0)	90.2 (89.5–90.9)	53.1	87.1	3.9	1.8
AMD_mortality_adults	170	HW	0.75	94.4 (93.9–94.9)	93.7 (93.7–93.8)	65.4	88.1	2.5	1.6
AMD_mortality_young	130.4	EWMA	0.75	95.5 (95.0–95.9)	95.7 (95.7–95.8)	77.4	90.6	1.7	1.6
ASR_GI_adults	125	HW	1.5	97.1 (96.7–97.5)	96.5 (96.1–96.9)	77.6	88.7	1.4	1.4
ALIS_BVD	105.4	HW	1	92.3 (91.6–92.8)	92.5 (91.9–93.1)	59.6	87.4	3.0	1.7
ALIS_IBR	94.8	EWMA	0.75	92.4 (91.8–93.0)	91.6 (91.5–91.7)	62.2	89.6	3.3	1.4
ALIS_abortion	79.6	HW	1.5	97.6 (97.3–98.0)	96.9 (96.5–97.3)	81.3	89.8	1.2	1.3
ASR_calves	36	HW	2	96.2 (95.7–96.6)	98.0 (98.3–98.8)	85.9	88.0	0.6	1.3
ASR_RESPI_adults	19	HW	2	97.4 (97.1–97.8)	98.5 (98.2–98.7)	87.3	89.0	0.6	1.0
ASR_RESPI_calves	17.7	HW	1	98.4 (98.1–98.7)	98.0 (97.7–98.3)	88.7	91.7	0.3	1.1
ASR_GI_calves	11	HW	2.25	98.9 (98.7–99.2)	99.0 (99.0–99.1)	93.3	89.7	0.4	1.2
ASR_Abortion	7	HW	3	99.4 (99.3–99.6)	99.4 (99.3–99.6)	95.7	89.3	0.2	1.2
ASR_OTHER_adults	6	HW	3	99.9 (99.8–99.9)	99.8 (99.7–99.9)	98.3	90.1	0.1	1.0
ASR_OTHER_calves	2.6	HW	3.5	99.5 (99.3–99.7)	99.7 (99.5–99.8)	97.4	89.7	0.1	0.9

*The optimal alarm threshold is defined as a multiple of the standard error. FP/yr, mean number of false positive alarms per year; T, timeliness in weeks; Se, Sensitivity; Sp, specificity; PPV, the positive predictive value; NPV, the negative predictive value. TS were ordered according to the weekly average number of cases from the largest (top row) to the smallest (bottom row)*.

**Table 4:** Detection performances obtained with the optimal algorithm at the optimal alarm threshold.

**Time series**	**Very small epidemics (25)**				**Small epidemics (50)**				**Medium epidemics (150)**			
	**Se**	**Sp**	**T**	**CC**	**Se**	**Sp**	**T**	**CC**	**Se**	**Sp**	**T**	**CC**
AMD_mortality_calves	60.6	89.1	2.5	24	71.6	89.3	2.6	36	97.1	89.9	2.4	90
ASR_adults	78.8	86.7	3.6	33	88.1	87.6	3.0	40	100	90.0	2.1	80
AMD_Stillbirth	75.1	88.1	3.0	28	87.0	88.6	2.7	40	100	89.7	1.6	68
AMD_mortality_adults	78.3	92.0	2.9	19	93.8	92.4	2.7	38	100	93.5	1.4	63
AMD_mortality_young	80.2	94.4	3.1	20	97.4	94.8	2.5	34	100	95.7	1.3	67
ASR_GI_adults	87.0	94.1	3.0	21	98.0	95.2	2.3	29	100	97.6	1.0	60
ALIS_BVD	71.8	88.8	2.6	17	89.6	89.9	2.8	38	100	93.1	1.6	75
ALIS_IBR	71.7	89.7	2.1	14	90.4	90.3	2.0	33	100	91.7	1.5	71
ALIS_abortion	88.0	94.0	2.9	20	99.0	96	2.3	29	100	97.9	1.0	58
ASR_calves	83.1	96.9	3.5	23	98.0	98.2	3.0	40	100	99.2	1.3	66
ASR_RESPI_adults	87.8	96.8	2.9	18	99.4	98.2	2.5	31	100	99.1	1.0	58
ASR_RESPI_calves	91.9	95.5	2.8	18	100	97.9	3.0	35	100	98.8	1.4	67
ASR_GI_calves	99.9	92.8	3.2	21	100	94.9	2.0	27	100	95.7	0.8	62
ASR_Abortion	97.0	99.3	3.2	22	100	99.4	2.0	30	100	99.4	0.6	55
ASR_OTHER_adults	99.2	99.7	3.1	21	100	99.7	1.8	28	100	99.7	0.4	54
ASR_OTHER_calves	97.4	99.6	2.7	17	100	99.6	1.5	23	100	99.6	0.3	59

*Se, Sensitivity; Sp, specificity; T, timeliness in weeks; CC, cum_cases, cumulative number of cases occurring because of the epidemic when the first alarm was raised. Results obtained for Large and very large epidemics are not shown as the detection performances were similar to those obtained for medium epidemics. TS were ordered according to the weekly average number of notifications from the largest (top row) to the smallest (bottom row)*.

**Figure 5:**
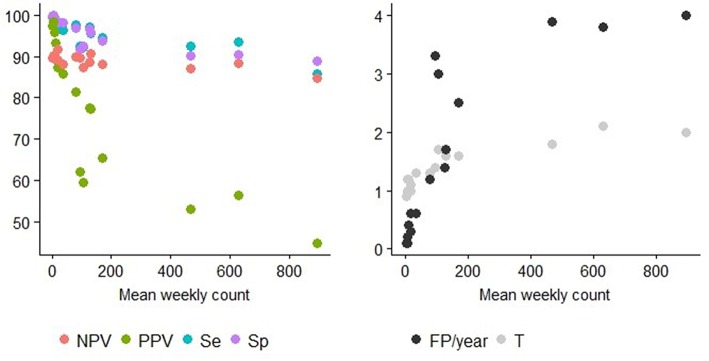
Detection performance at the optimal alarm threshold and Mean weekly count. Y axis of the left graph: percentage of specificity (Sp), sensitivity (Se), positive predictive value (PPV), or the negative predictive value (NPV). Y axis of the right graph: number of false positive alarms per year (FP/year) or number of weeks before the first true positive alarm is raised (T = detection timeliness). The different TS can be distinguished using the information provided in Table 2.

Syndrome TS with lower mean weekly counts (e.g., *ASR_RESPI_calves, ASR_GI_calves, ASR_abortion, ASR_OTHER_adults, ASR_OTHER_calves*) were better for detecting all epidemics, as there was a general increase in the overall detection performance for all metrics as the mean weekly count in the syndrome TS decreased (Table 3). However, the relationship varied between metrics. *Se* and *Sp* decreased in syndrome TS with the largest mean weekly counts (Spearman ρ coefficients equal to 0.878; *P* < 0.0001 for *Se*, and 0.941; *P* < 0.0001 for *Sp*). PPV had the strongest relationship with syndrome TS mean weekly count. The smaller the mean weekly count the larger the PPV ranging from 44.9 to 97.4 in syndrome TS with the largest to smallest mean weekly counts, respectively (Spearman ρ coefficient 0.95; *P* < 0.0001) (see Figure 5). The average number of false positive signals per year decreased for syndrome TS with the largest to the smallest mean weekly counts, respectively (Spearman ρ coefficient −0.945; *P* < 0.0001). Timeliness decreased when the weekly mean number of counts increased (Spearman ρ coefficient −0.943; *P* < 0.0001). NPV had the weakest relationship with syndrome TS mean weekly count (Spearman ρ coefficient 0.610; *P* = 0.012). The observation that increased detection performance was associated with decreased mean weekly counts was not related to decreasing relative variance in syndrome TS with smaller weekly mean counts. The CV increased as the mean weekly count of the syndrome TS decreased (Spearman ρ coefficient −0.863; *P* < 0.0001) (see Figure 5).

Small epidemics were detected earlier on average than very small epidemics but with a higher number of cumulative cases. This is consistent with the method used for simulating the epidemics. The three syndrome TS with the largest weekly baseline counts (i.e., *AMD_mortality_calves, ASR_adults, AMD_stillbirth*) tended to detect small and very small epidemics later and with a higher average cumulative number of cases at the time of detection than syndrome TS with smaller weekly baseline counts. Only half of the syndrome TS were able to detect very small epidemics (i.e., magnitude 25) with a sensitivity above 85%. However, all syndrome TS except *AMD_mortality_calves* detected more than 85% of the small epidemics (i.e., magnitude 50). Time-series with high weekly counts were very poor for detecting very small and small epidemics. Only syndrome TS with small weekly counts detected more than 90% of the very small epidemics. The syndrome TS with the highest average number of reports per week, *AMD_mortality_calves*, detected only 60.6% of these epidemics. Only the syndrome TS with <200 counts per week on average could detect more than 90% of the small epidemics

To test the theory that changing the alarm threshold may increase detection performance, we modified the alarm threshold for the 11 aberration detection algorithm-syndrome TS pairs that did not detect more than 90% of the very small epidemics. The smallest alarm threshold able to provide a sensitivity for very small epidemics equal to or above 90 % was defined as the optimized alarm threshold. The new detection performances obtained with this optimized alarm threshold are presented in Table 5. Most of the syndrome TS were able to detect more than 90% of the very small epidemics by using the optimized alarm threshold. However, three time series *AMD_mortality_calves, ASR_adults*, and *AMD_stillbirth* were never able to reach this level of detection performance even when using a very low alarm threshold (i.e., 0.05 times the standard error of the prediction). In addition, increasing the sensitivity for very small epidemics reduced the overall specificity of the detection. For example the specificity of *AMD_Stillbirth* dropped from 90.2 to 82.1% when the alarm threshold changed from 0.5 to 0.05 times the standard error of the prediction, resulting in more than 7 false alarms per year.

**Table 5:** Detection performance obtained with the optimal algorithm at the optimized alarm threshold.

**Time series**	**Optimal algorithm**	**Optimized alarm threshold**	**Overall Se**	**Overall Sp**	**Very small epidemics (25)**		**Small epidemics (50)**	
					**Se**	**Sp**	**Se**	**Sp**
AMD_mortality_calves	HW	0.05	85.9	89.8	60.6	89.1	71.6	89.3
ASR_adults	HW	0.05	96.3	86.4	87.2	82.5	94.5	83.6
AMD_Stillbirth	HW	0.05	96.3	82.1	87.1	79.3	94.6	80.1
AMD_mortality_adults	HW	0.05	98.0	83.8	91.8	80.9	98.3	81.6
AMD_mortality_young	EWMA	0.5	98.6	88.5	93.6	85.8	99.7	87.0
ASR_GI_adults	HW	1	98.4	93.0	93.0	89.4	99.2	90.7
ALIS_BVD	HW	0.5	96.7	87.4	86.5	82.4	97.0	83.6
ALIS_IBR	EWMA	0.25	98.7	79.2	94.0	74.9	99.6	76.3
ALIS_abortion	HW	1	99.2	93.8	96.1	89.4	100	92.1
ASR_calves	HW	1.5	98.4	97.1	92.5	94.6	99.8	96.3
ASR_RESPI_adults	HW	1.5	99.0	97.3	95.4	95.0	100	96.6

*The optimized alarm threshold is define as a multiple of the standard error. Se, Sensitivity; Sp, specificity*.

## Discussion

In this study, different syndrome TS performed differently depending on the type and magnitude of simulated epidemic, suggesting that all syndrome TS are not equally suited for detecting all types and magnitudes of epidemics. Our study illustrates that the event detection performance is dependent on the characteristics of three components: the syndrome TS, the epidemic, and the aberration detection algorithm. Since these three components are interrelated, they should be evaluated together.

The two detection algorithms used in this study were selected because they are easy to automate and they can be implemented on short baseline TS and (30). We expected to see differences in detection performance between the two algorithms for the different epidemic shapes. The EWMA algorithm has been reported to perform well for detecting small but repeated differences between observed and expected values, as seen in flat or linear epidemics (16, 21, 23). Holt-Winters method has been reported to be more effective for detecting large epidemics with a sudden increase in cases such as in single peak or exponential epidemics (22, 23). These performance differences were not supported by the results of our study. Somewhat unexpectedly, we identified optimal algorithms for each syndrome TS that performed equally well across all epidemic shapes. The HW algorithm outperformed the EWMA algorithm for most of the syndrome TS (i.e., 14 out of 16 syndrome TS) which confirms reports from previous studies (23, 27). However, the EWMA algorithm outperformed the HW algorithm on the *AMD_mortality_young*, and *ALIS_IBR* syndrome TS. This may partially be explained by the high mean weekly counts in these syndrome TS combined with the poor data forecasting performance of the HW algorithm. The latter may be at least partly due to the complex temporal patterns observed in these syndrome TS (see Table 2 and Figure 1) which has been reported to make the HW algorithm less well-adapted to TS (31). There are many other methods available for aberration detection and some of these could also be used for TS selection [see for example (31)]. Adding information about the total cattle population under surveillance and working with proportions instead of count data could also be tested to take into account shifts in submissions and possibly improve detection performance. In addition, testing different values of the smoothing parameters for the EMWA algorithm could be explored for improving detection performance. However, longer historical syndrome TS would be needed to develop better models, especially for syndrome TS in the ALIS database which had <3 years of data.

In our study, overall detection performance (collated for all epidemic types) differed greatly from individual detection performance for epidemics of different magnitudes. It was not surprising that detection performance was lower for small and very small epidemics compared to larger epidemics. Small increases in cases per unit of time can easily remain unnoticed in the background noise of a TS, especially when the TS contains on average, a large numbers of cases per unit of time. Other studies have assessed TS detection performance using epidemics of different magnitude, but only reported overall detection performance by collating the results obtained for epidemics of different magnitude [see for example (17, 24)]. Our study demonstrated that overall detection performance may result in misleading interpretations of the sensitivity and specificity of the surveillance system. Overall detection performance may mask the fact that a specific detection algorithm applied to a specific TS may actually only detect large increases in the number of cases. For example, *AMD_mortality_calves* syndrome TS performed very well overall (Sensitivity = 85.9 and specificity = 89.9 for all epidemics combined) but performed poorly for detecting very small and small epidemics, as only 60.6% of the very small epidemics and 71.6% of the small epidemics were detected. We strongly recommend that in future studies researchers report the specific detection performance obtained for different epidemic sizes and shapes in order to avoid overestimating the overall detection performance of the surveillance system.

In our study, small epidemics remained largely unnoticed in certain syndrome TS, especially when the mean baseline count, and the background noise were high (e.g., syndrome TS extracted from AMD). Adjusting the alarm threshold is a strategy for increasing sensitivity, but it increases the number of false alarms. Increasing the number of false alarms is a problem when monitoring several syndrome TS at the same time, as surveillance systems monitoring multiple syndromic TS have been reported to intrinsically suffer from a lack of specificity (32). An alternative approach to improve detection performance is to split large syndrome TS into smaller sub-TS or, in other words, to change the level of TS clustering. If syndrome TS are sufficiently large, splitting them into sub-TS can reduce the background noise in the sub-TS. This will increase the ratio of “epidemic cases” to “baseline cases,” and potentially improve detection performance. But only if the epidemic cases are not expected to be split among the sub-TS. For example, when geographical information is available, hierarchical time series approaches (33) or other spatiotemporal methods [see for example (34, 35)] could be used to improve detection performance as epidemics of transmissible diseases are supposed to start in a localized geographical area. When the epidemic is expected to be split among the sub-TS (e.g., when splitting a syndrome TS according to the production type and when all production types are susceptible to the disease), the benefit of splitting the data may be reduced. Splitting the data into different sub-TS should be carefully discussed as the benefit in terms of improved detection performance may not always offset the extra effort needed to properly monitor additional TS. Monitoring syndrome TS with low counts also has disadvantages. There is an increased risk of producing excessive numbers of false positive alarms (24), especially when the mean count per time unit is ≤ 5 (36, 37).

Syndrome TS that do not perform well for detecting small epidemics may have other uses in surveillance (8). SyS data can be used to define the normal behavior of disease and pathogens in animal populations in the absence of a specific epidemic. This information may have value for setting national benchmarks (38) or for supporting other surveillance programs (39). SyS may provide some evidence for the absence of certain diseases, or it may help to better understand farmers' production practices and veterinarians' clinical practices. Bovine Virus Diarrhea and IBR in Switzerland and the associated syndrome TS (*ALIS_BVD* and *ALIS_IBR)* are a good illustration of the potential alternative use of syndrome TS. Switzerland started an eradication program for BVD in 2008 which dramatically reduced the number of BVD cases (40) and the country has been officially free from IBR since 1990 (41). In our study, both syndrome TS had poor detection performance. Using other syndrome TS alone or a combination of syndrome TS, could potentially be more effective for early detection of a new epidemic of BVD or IBR in Switzerland. The syndrome TS *ALIS_BVD* and *ALIS_IBR* may have more value for monitoring long term trends in the epidemiological situation of the two diseases. This information could be especially relevant for BVD, as Switzerland is not free from the disease. The *ALIS_BVD* syndrome TS could be used to monitor long term trends in the number of suspect BVD cases, which may be useful for monitoring the impact of control measures, or farmer and veterinarian responses to these control programs.

The epidemics used in our study were simulated as vectors containing a fixed number of extra epidemic cases, which were added to all epidemic free baseline syndrome TS. This method proposed by Lotze et al. (25) was chosen because it allows the creation of standardized simulated epidemics that are constant for all syndrome TS being evaluated. Using standardized epidemics allows for the direct comparison of the performance of different syndrome TS. For example, a “small” epidemic will have the same number of epidemic cases when it is inserted into either a small (having a small mean number of cases per unit of time) or large (having a large mean number of cases per unit time) baseline syndrome TS. The size of the inserted epidemic will also be constant for baseline syndrome TS, which have small or large variation in the number of cases per unit of time. The other commonly reported method for epidemic simulation defines the number of epidemic cases as a multiple of the standard deviation of the baseline syndrome TS [see for example (17, 24, 25, 42)]. Both approaches are perfectly suitable for epidemic simulation and the choice of one or the other depends on user preferences (25). However, the second method may not be as easy to use for the direct comparison of syndrome TS that have different standard deviations. Difficulty arises because the size of the simulated epidemics inserted into syndrome TS with different standard deviations will not be the same. For example, a simulated epidemic with a magnitude 2 times the standard deviation will produce 20 epidemic cases for a syndrome TS with a standard deviation of 10, and 200 epidemic cases for a syndrome TS with a standard deviation of 100. Computationally the two methods are comparable because transforming a multiple of the standard deviation into the corresponding number of extra cases and vise versa is quite straightforward. However, interpreting epidemics based on multiples of standard deviations is more difficult and may in some situations result in misleading interpretations of detection performance. For example, consider the case where an algorithm has been shown to detect more than 90% of small simulated epidemics and where the small simulated epidemic magnitude equals 2 times the standard deviation of the syndrome TS being evaluated. This detection performance may appear sufficient for detecting small epidemics, but if the standard deviation of the syndrome TS was quite large, it could mean that only large epidemics were being detected. When the objective of syndrome TS evaluation is to operationalize a SyS system for field use, we recommend the approach used in our study, where each type of simulated epidemic has a constant number of cases for all syndrome TS being evaluated. This approach more closely resembles field situations where we expect the size of an epidemic to have no relationship to the standard deviation of a syndrome TS. It also closely aligns with the way that surveillance practitioners characterize epidemics, which is by counting cases to map epidemic growth and geographic spread. They do not characterize epidemic growth in terms of increases in the number of standard deviations of the baseline case TS.

The objective of the SyS system currently being developed in Switzerland is to detect an epidemic of any disease in the Swiss cattle population. Based on our approach and results, most of the syndrome TS considered in this study may have value for this SyS system. Indeed, it was possible to accurately and timely detect small changes occurring in most of the syndrome TS considered. Our results also indicated that some syndrome TS should be excluded from an early detection SyS system because of their poor detection performance. This is the case for the syndrome TS *ALIS_IBR* and *ALIS_BVD*. However, they may have value for other surveillance purposes. The syndrome TS extracted from the AMD dataset also performed poorly and their usefulness for early epidemic detection is questionable. Except for the *AMD_stillbirth*, syndrome TS, all other syndrome TS from the AMD dataset consisted of counts of cattle mortalities. Cattle mortalities may not be the best indicator for early disease detection. To obtain a detectable signal in these syndrome TS, the excess mortality from an epidemic in the population would have to be high. High mortality is easily noticed by veterinarians or farmers and would likely be reported through traditional passive surveillance. Smaller epidemics caused by diseases with low mortality could remain unnoticed or signals may not be generated in these syndrome TS until late in the course of an epidemic. However, cattle mortality syndrome TS may be of interest for investigating the consequences of an epidemic. The objective of our study was to present a method that surveillance practitioners could use to help select syndrome TS-event detection pairs for inclusion in a surveillance system. The method estimates the minimum size of various types of simulated epidemics that could potentially be detected in syndrome TS-event detection pairs. We wish to point out that this is not the only evaluation criterion that should be used to select TS for inclusion in a SyS system. Before drawing any final conclusion regarding which syndrome TS to include in the Swiss SyS system, other selection criteria such as the representativeness and quality of the data should be carefully considered (10, 43–45). For example, the ASR data that we used did not contain data about the Swiss beef cattle population, which might reduce the benefit of this data sources for disease early detection. Assessing the population coverage of this data source would be essential before including this data source in a national surveillance system. The lack of consistency in the definition of stillbirth in the AMD data may also be an issue and might lead to inconsistent data reporting. We recommend a holistic approach that considers all TS characteristics. The criterion “the minimum size of event that could be detected in syndrome TS-event detection pairs” that we presented in this study should be only one of the criteria considered. In addition, in this study TS were evaluated individually but in future studies it would be interesting to evaluate TS together using multivariate aberration detection algorithms.

## Conclusion

Our study results demonstrate that syndrome TS are not all of equal value for early epidemic detection. Event detection performance is dependent on the characteristics of the syndrome TS, the nature of the epidemic being targeted, and the event detection algorithm. Final selection of specific syndrome TS for inclusion in an operational SyS system will be dependent on the performance characteristics of the syndrome TS and also on the goals of the surveillance initiative. It is not possible to set specific decision rules that can apply to all situations. However, the results of our study suggest that surveillance system designers should carefully assess each candidate syndrome TS before including it in their early epidemic surveillance system. The assessment should include fitting an optimal event detection algorithm to the syndrome TS and then evaluating the detection performance of the syndrome TS-algorithm pair on a variety of epidemic types. Only those syndrome TS which have acceptable performance for epidemics types that are similar to epidemics of the disease under surveillance should be included in the SyS system. Evaluating the ability of syndrome TS for early detection of epidemics is essential for selecting syndrome TS for a syndromic surveillance system, as early epidemic detection is the central task of syndromic surveillance.

## Data Availability Statement

The datasets for this study will not be made publicly available because the data contain confidential information on Swiss farms that cannot not be shared with a third party.

## Author Contributions

CF and JB designed the study. CF performed the analysis. CF, SS, and DH provided access to the data. All authors wrote and reviewed the paper.

### Conflict of Interest

The authors declare that the research was conducted in the absence of any commercial or financial relationships that could be construed as a potential conflict of interest.

## Footnotes

^1^See http://www.blv.admin.ch/gesundheittiere/03007/index.html?lang=en
