# Supplementary material for: Simulation Based Evaluation of Time Series for Syndromic Surveillance of Cattle in Switzerland
Source: Front Vet Sci. 2019 Nov 5;6:389. doi: 10.3389/fvets.2019.00389 (PMC6856673; doi:10.3389/fvets.2019.00389)
Supplement: Supplementary file 1 [file Data_Sheet_1.docx]

**Supplementary information with**

**“Simulation based evaluation of 16 time series for syndromic surveillance of cattle in Switzerland”**

**APPENDIX 1: Epidemic simulation**

**APPENDIX 2: Time series modelling**

2.A: Results of TS modelling and prediction using HW

2.B: Autocorrelation in the TS after the 1-week differencing

2.C: ACF, PACF of the raw TS

**APPENDIX 3: Aberration detection performance**

**APPENDIX 4: Evaluation of the optimal alarm threshold**

**APPENDIX 1: Epidemic simulation**


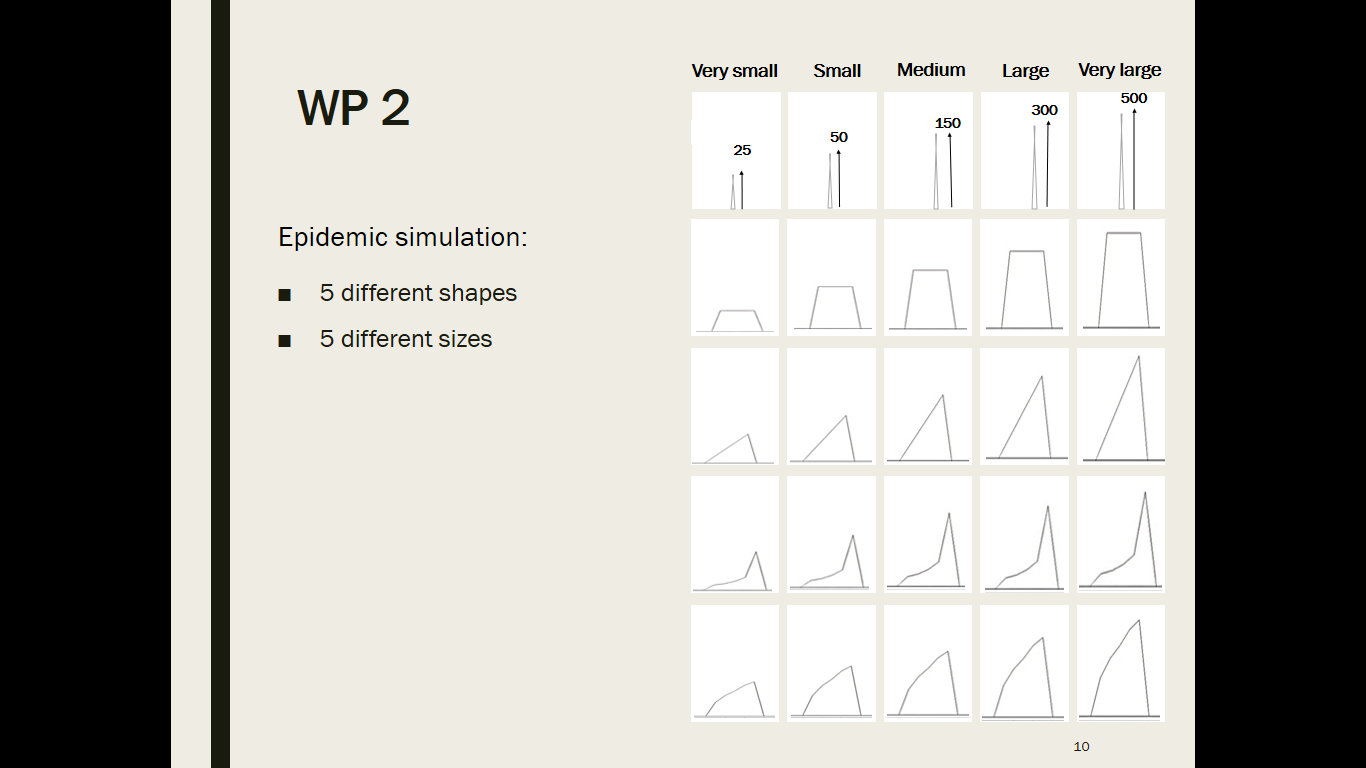


**Log normal increase**

**Flat increase**

**Linear increase**

**Single peak**

**Exponential increase**

Figure 1: Different shape and approximate size of the 25 different type of epidemic simulated

**APPENDIX 2: Time series modelling**

2.A: Results of time series modelling and prediction using HW


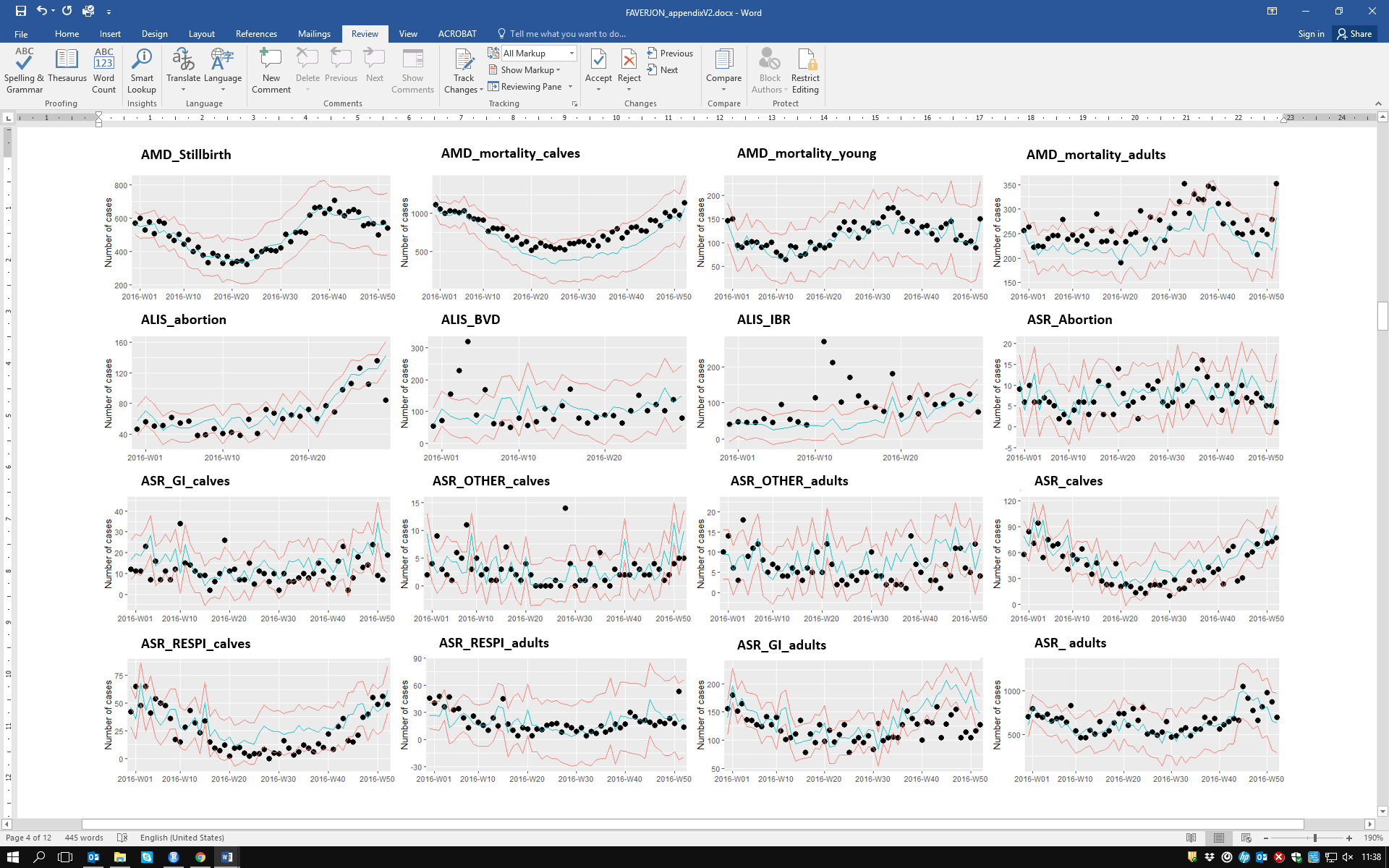


Figure 1: Prediction performance of HW for the 16 TS. Blue lines = mean fitted value, red lines = 95% confidence interval (CI), black dots = real count data

| Time series | RMSE_t_ | RMSE_v_ |
| --- | --- | --- |
| AMD_mortality_calves | 70.7 | 128.7 |
| ASR_adults | 69.1 | 117.0 |
| AMD_stillbirth | 35.4 | 40.8 |
| AMD_mortality_adults | 18.8 | 35.6 |
| AMD_mortality_young | 22.0 | 43.0 |
| ASR_GI_adults | 15.4 | 34.7 |
| ALIS_BVD | 28.3 | 66.7 |
| ALIS_IBR | 43.2 | 21.3 |
| ALIS_abortion | 9.5 | 21.1 |
| ASR_calves | 9.4 | 17.2 |
| ASR_RESPI_adults | 7.4 | 17.8 |
| ASR_RESPI_calves | 9.8 | 17.4 |
| ASR_GI_calves | 4.9 | 8.5 |
| ASR_abortion | 3.1 | 4.0 |
| ASR_OTHER_adults | 3.1 | 5.6 |
| ASR_OTHER_calves | 2.1 | 3.2 |

Table 1: Root-mean-squared error for the training (RMSE_t_) and validation period (RMSE_v_)


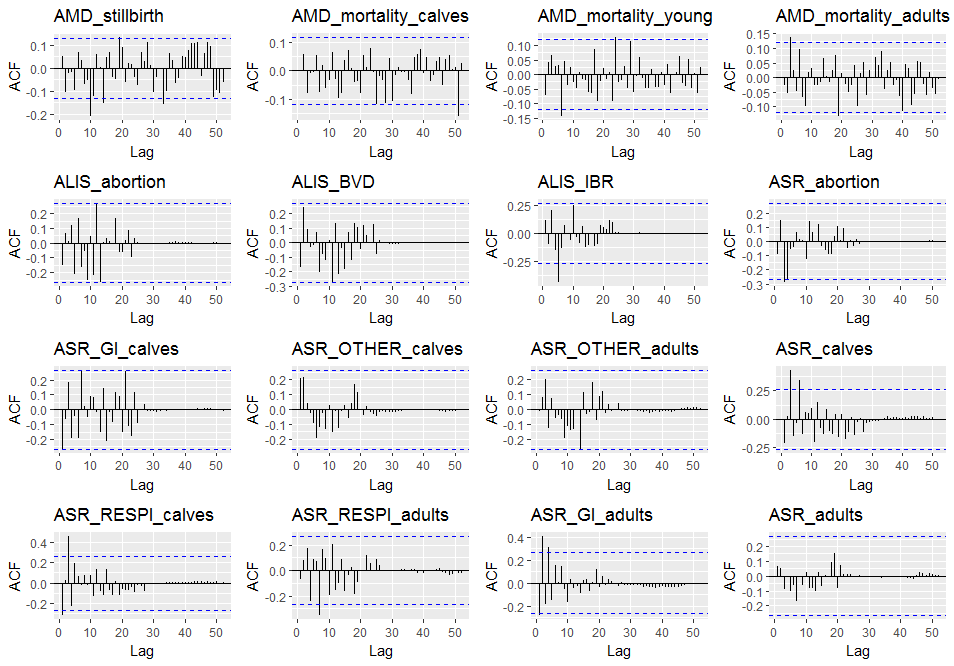


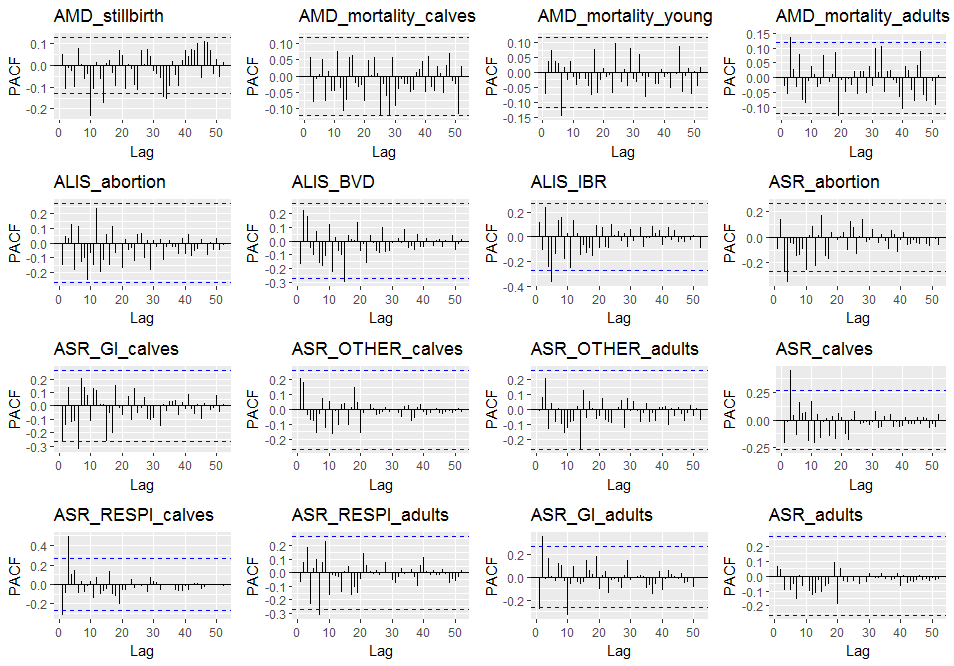


Figure 2: Autocorrelation (ACF) and Partial Autocorrelation (PACF) of the residuals obtained with the training dataset


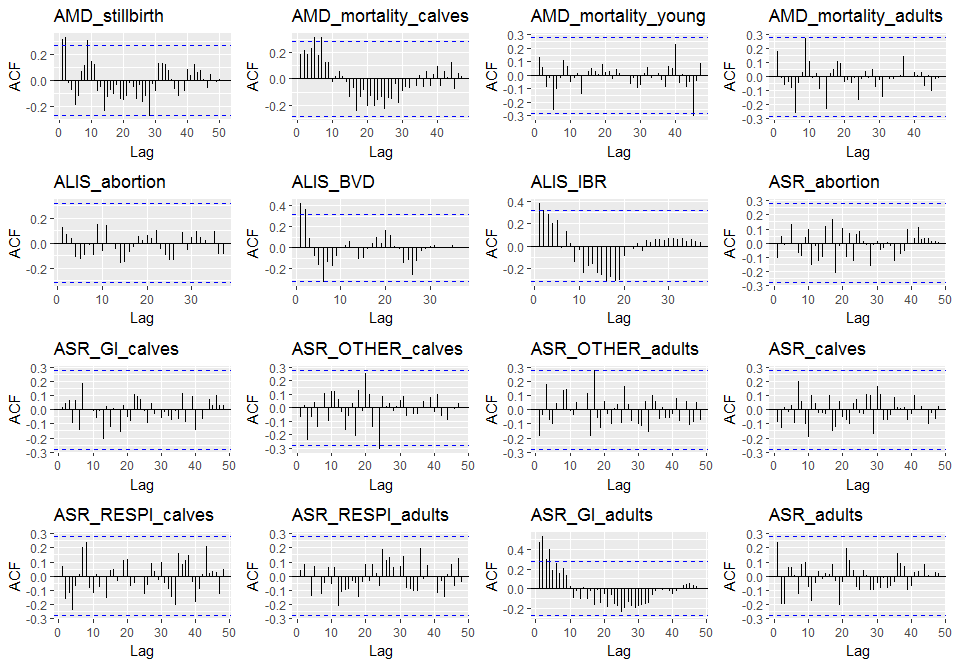

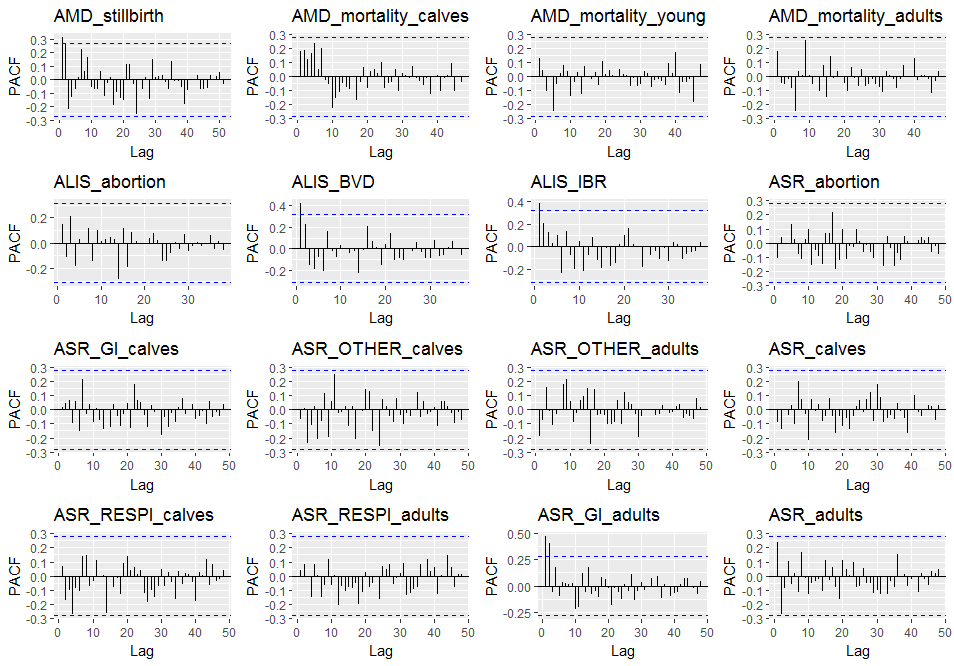


Figure 3: Autocorrelation (ACF) and Partial Autocorrelation (PACF) of the residuals obtained with the validation dataset

2.B: Autocorrelation in the time-series after 1-week differencing


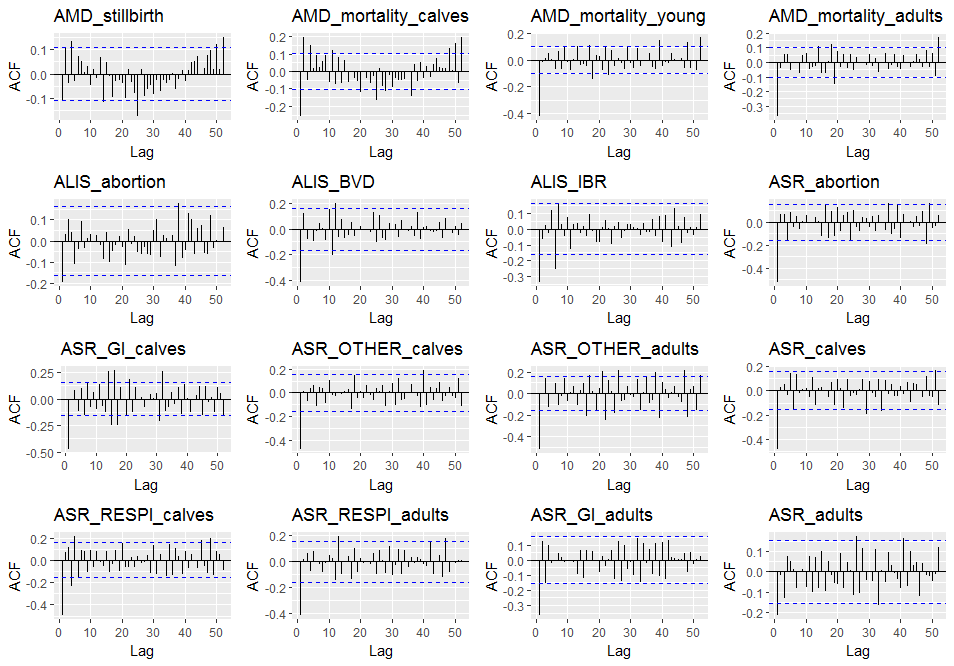

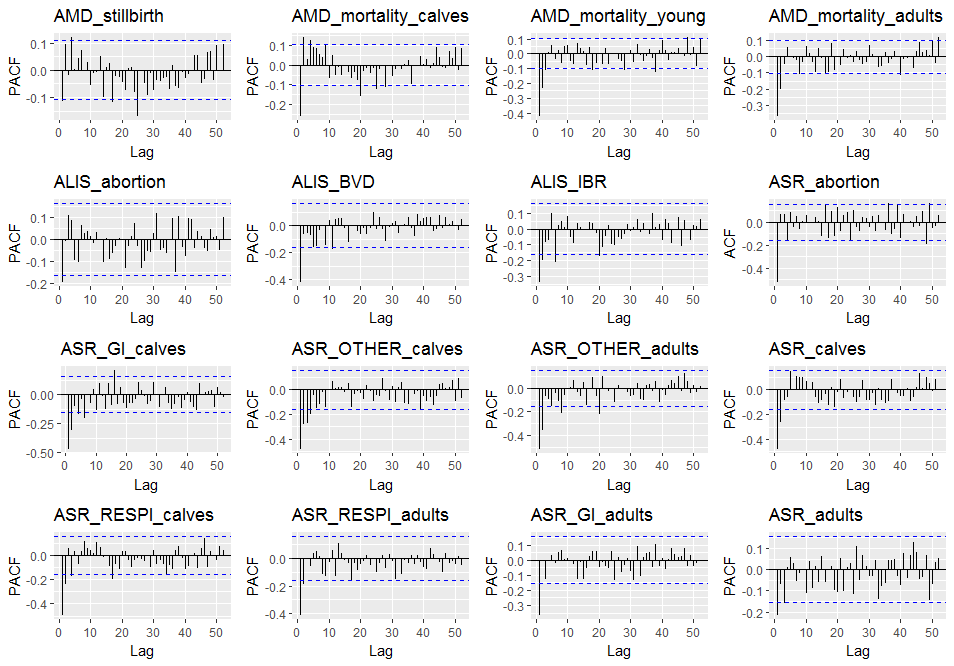


Figure 1: Autocorrelation (ACF) and Partial Autocorrelation (PACF) of the data after 1-week differencing

2.C: Autocorrelation in the raw time-series


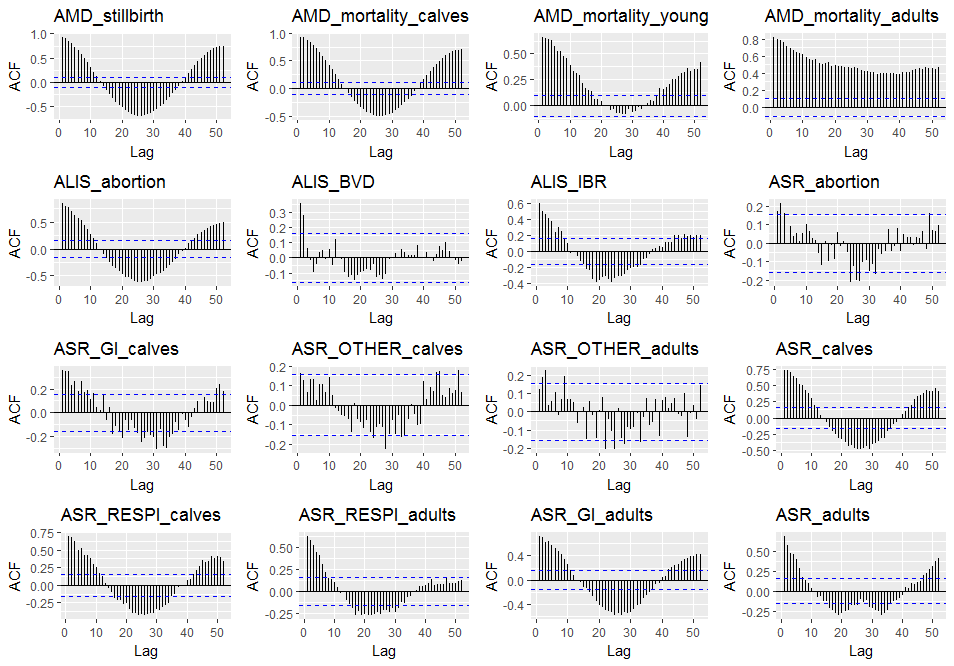

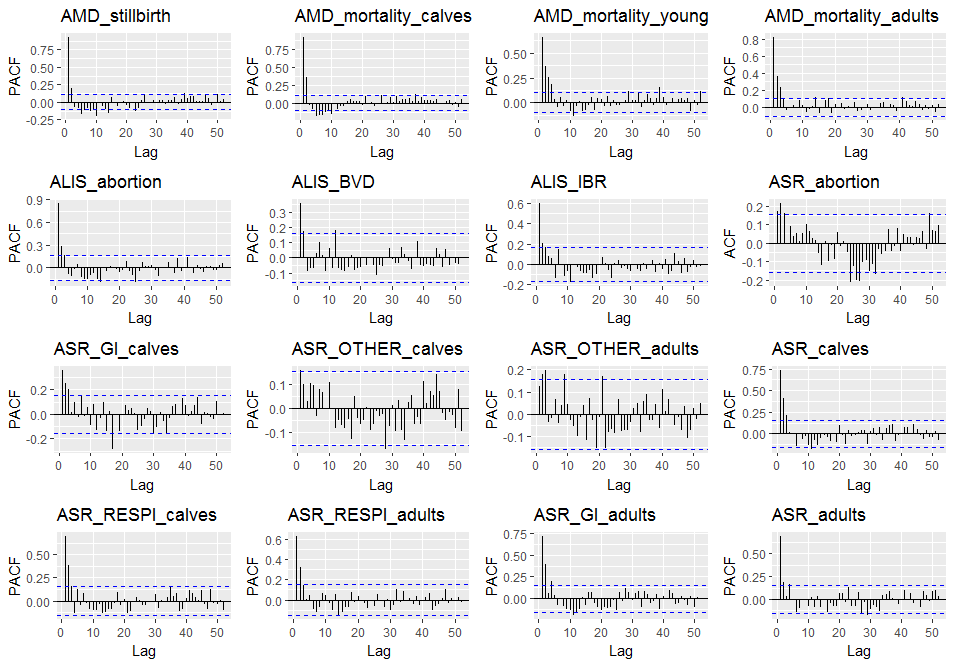


Figure 1: Autocorrelation(ACF) and Partial Autocorrelation(PACF) of the raw data

**APPENDIX 3: Aberration detection performance**


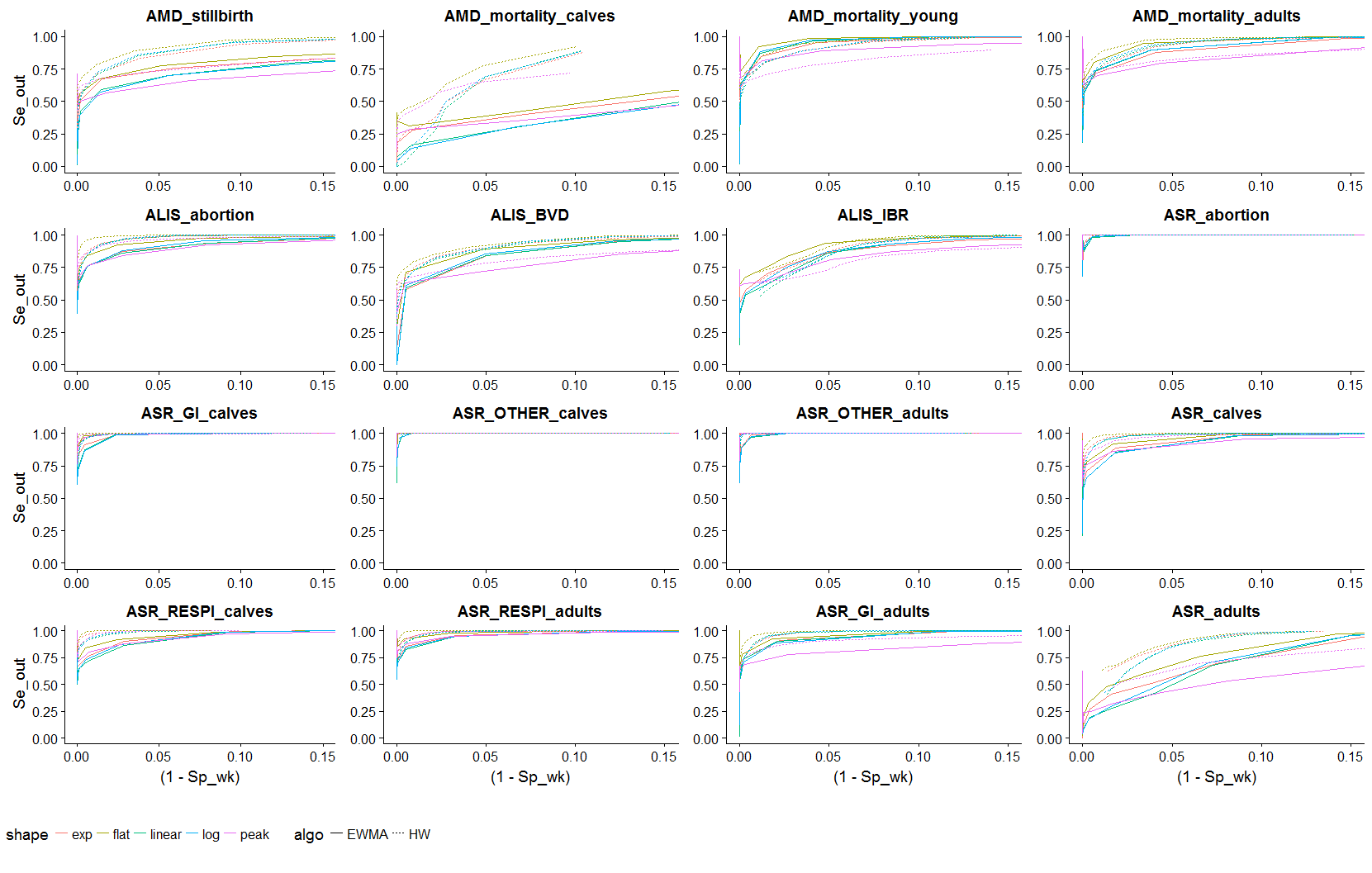


Figure 1: Receiver-operating characteristic (ROC) curve for the 16 syndrome TS, the 2 aberration detection algorithms and the 5 different outbreak shapes (all outbreak magnitudes collated).


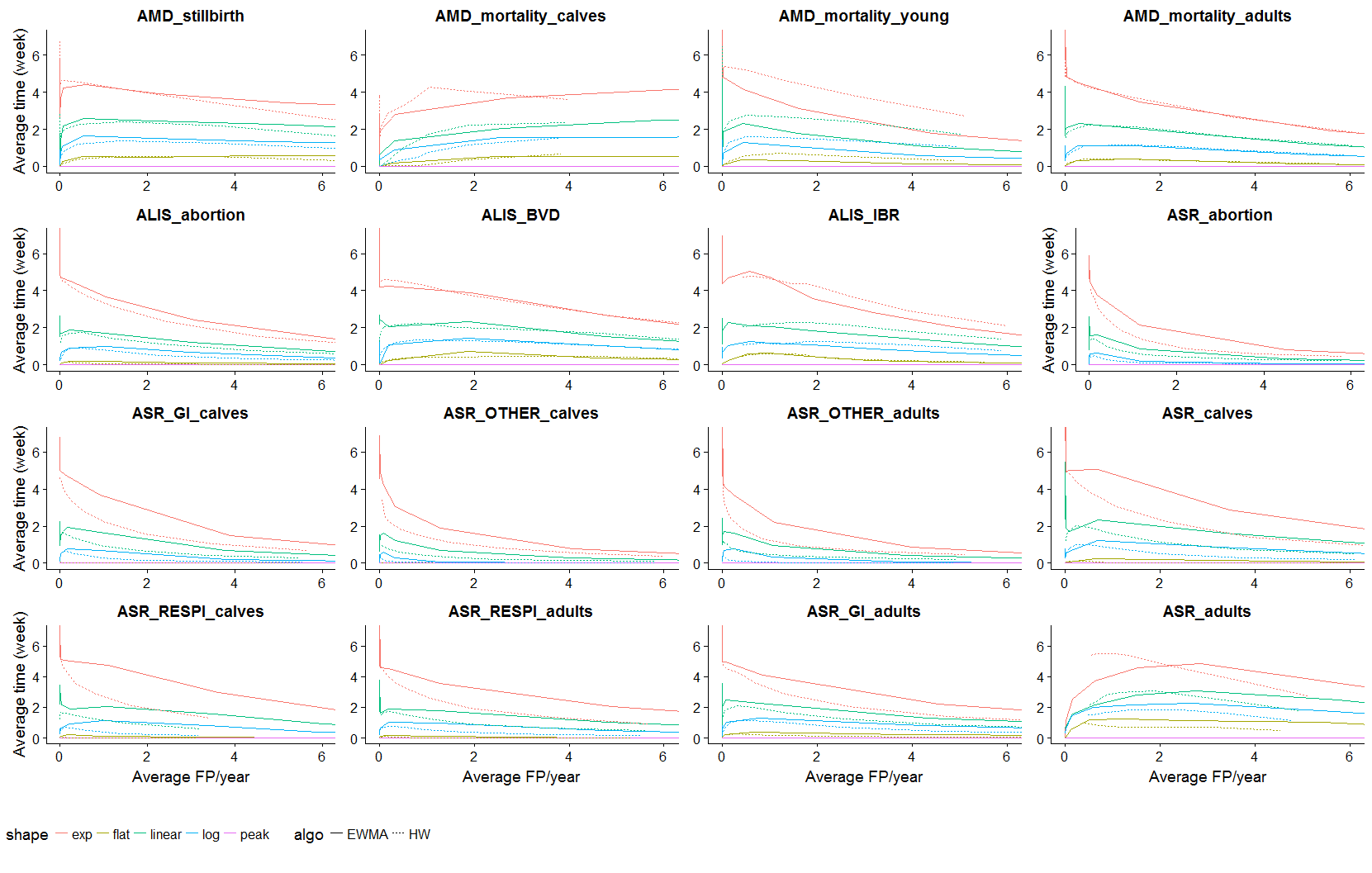


Figure 2: Overall average detection time (week) and corresponding average number of expected false alarms per year for the 16 syndromes, the 2 aberration detection algorithms and the 5 different outbreak shapes (all outbreaks magnitudes collated).

**APPENDIX 4: Evaluation of the optimal alarm threshold**

**
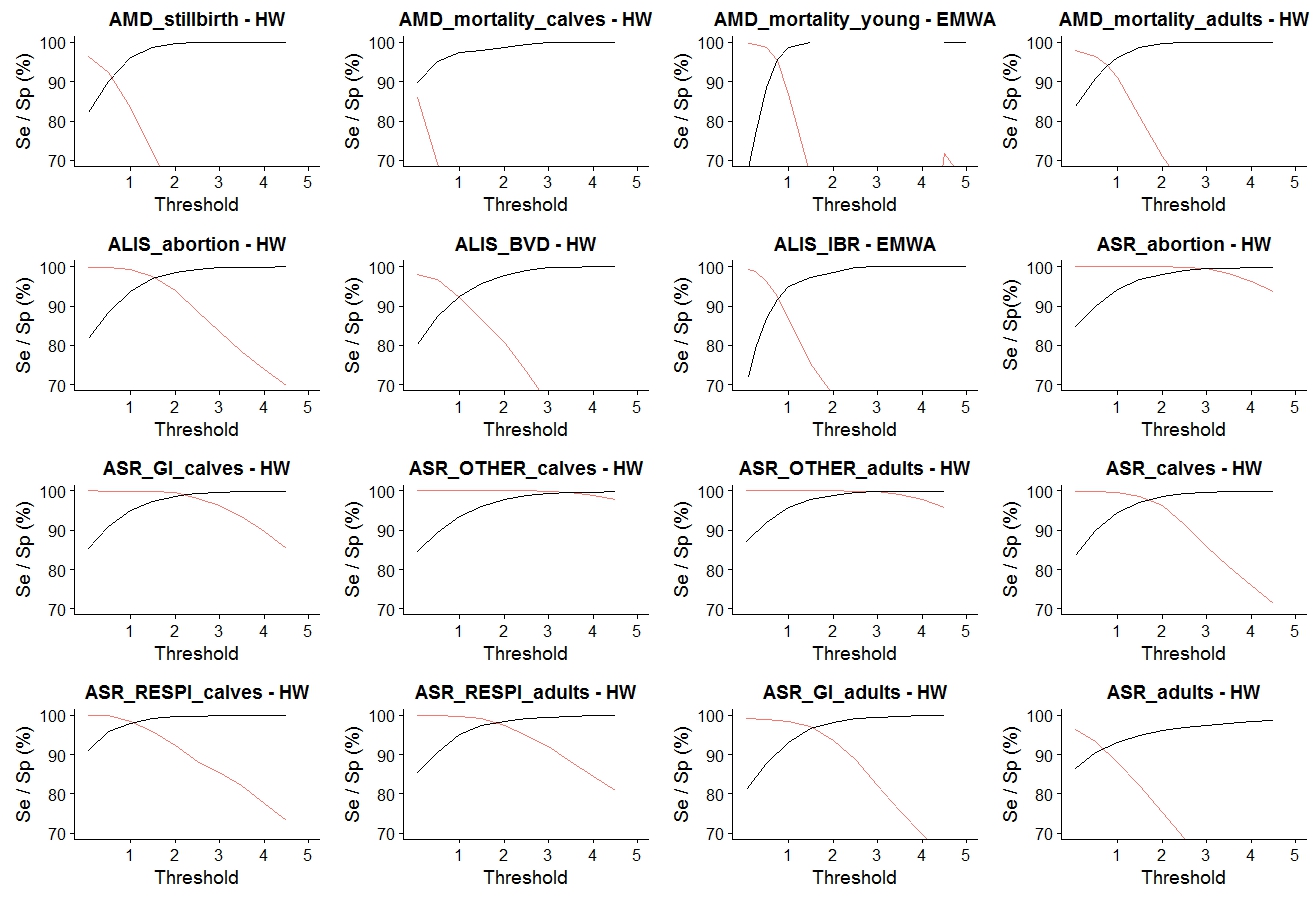
**

Figure 1: Evaluation of the optimal alarm threshold for each TS used with its optimal algorithms. Assuming equal costs for false negative and false positive alarms, we graphically defined the optimal alarm threshold where *Se* and *Sp* were at a maximum (i.e., where the red and black lines crossed)
